# Probiotics and Ozonated Olive Oil to Maintain Oral Eubiosis in Stage I and II Periodontitis Patients: A Randomized Triple-Blind Clinical Trial

**DOI:** 10.3390/dj14040203

**Published:** 2026-04-01

**Authors:** Antonia Abbinante, Giuseppe Barile, Anna Antonacci, Matteo Basso, Francesca Pascale, Nicola Bartolomeo, Maria Teresa Agneta, Giuseppe D’Albis, Tommaso Corsalini, Saverio Capodiferro, Massimo Corsalini

**Affiliations:** 1 Department of Interdisciplinary Medicine, Università degli Studi di Bari ‘Aldo Moro’, 70121 Bari, Italy; antonella.abbinante@gmail.com (A.A.); anna_antonacci@yahoo.it (A.A.); fra.pasc24@gmail.com (F.P.); nicola.bartolomeo@uniba.it (N.B.); agneta.mt.mta@gmail.com (M.T.A.); giuseppe.dalbis@uniba.it (G.D.); tommasocorsalini@gmail.com (T.C.); saverio.capodiferro@uniba.it (S.C.); massimo.corsalini@uniba.it (M.C.); 2 Lake Como Institute, Università degli Studi di Milano, 22100 Como, Italy; segreteria@studiobassoritzmann.it

**Keywords:** periodontitis, probiotics, ozonated olive oil

## Abstract

**Background:** Researchers are now focusing on new and less invasive therapies to improve the domiciliary maintenance phase of periodontitis. Ozonated olive oil as an alternative to common local antiseptics and the assumption of probiotics to maintain a eubiotic oral microbiome show promising results. However, the literature is still limited on this topic. This RCT aims to investigate the clinical benefits of combining ozonated olive oil products (mouthwash and toothpaste) with probiotics on oral health status in patients with stage I and II periodontitis following the active phase of therapy. **Methods:** The study followed a triple-blind RCT design. Patients with stage I and II periodontitis were enrolled and randomly assigned to three groups: group A (placebo), group B (effective ozonated olive oil mouthwash and toothpaste), and group C (combined protocol with effective ozonated olive oil and probiotics). Clinical assessment was performed at the first visit and after 30 days, considering Full-Mouth Plaque Score (FMPS), Full-Mouth Bleeding Score (FMBS), and Probing Pocket Depth (PPD). **Results:** The FMPS percentages showed a significative reduction (*p* = 0.0002) of 24%, 33%, and 62% observed in group A, group B, and group C, respectively. Also, the FMBS percentages were significantly decreased (*p* < 0.0001): −15%, −20%, and −49% observed in group A, group B, and group C, respectively. The mean PPD showed significant differences (*p* < 0.0001): −0.10 mm, −0.40 mm, and −1.10 mm observed in group A, group B, and group C, respectively. Overall, group C showed the best improvement among the considered clinical indexes. **Conclusions:** The findings of this clinical trial support the use of a combined regime of the antimicrobial and anti-inflammatory effects of ozonated olive oil and the modulation of the oral microbiome of probiotic supplements as an adjunctive domiciliary strategy for patients affected by stage I and II periodontitis.

## 1. Introduction

Periodontal diseases are chronic inflammatory conditions affecting the supporting tissues of the teeth, including the gums (gingivitis) and the deep periodontium (periodontitis). These diseases are primarily infectious in origin, resulting from interactions between pathogens (bacteria, viruses, yeasts) and the host’s immune system. According to the Global Burden of Disease Study, periodontal disease is rampant, with a global prevalence of 50% and a significant impact in Italy, affecting up to 60% of the population, with 10% of cases in advanced forms [1]. Today, there is extensive knowledge of the diagnosis and treatment of periodontal disease, underscoring the importance of a proper, customized maintenance plan, including regular check-ups and the prescription of domiciliary mechanical and chemical aids to prevent relapses. Periodontitis treatment follows a gradual approach, from controlling gingival inflammation and plaque to more complex interventions, such as periodontal surgery in advanced stages [2]. Moreover, the European Federation of Periodontology (EFP) guidelines recommend chlorhexidine mouth rinses for a limited period of time, and locally administered sustained-release chlorhexidine or other antibiotics as an adjunct to professional scaling and root planning therapies in stage I–III periodontitis [3]. Dysbiosis and the subsequent inflammatory process play a pivotal role in the recurrence of periodontitis, so an effective approach could interfere with the early recolonization of residual pockets and previously treated areas using dysbiotic microbiota [4,5,6]. The use of probiotics in patients affected by periodontitis aims to promote the early formation of less aggressive biofilms, thereby maintaining eubiosis, which is particularly important for patients with risk factors that may lead to disease relapse [7]. Many drugs and devices with proven effectiveness promote eubiotic microbiota: probiotics, antiseptics, dietary supplements, gaseous ozone, or ozonated oils are just a few examples [8,9]. Adequate support for domiciliary therapy is essential to maintain periodontal stability and prevent recurrence. Chlorhexidine is the most commonly used antiseptic agent in the maintenance therapy of periodontitis, as supported by the recent literature [10]. It is characterized by its broad spectrum of action, effectively exerting bacteriostatic and bactericidal effects against many Gram-positive and Gram-negative bacteria, including Porphyromonas gingivalis, Bacteroides forsythus, Actinomyces, Actinomyces comitans, and Prevotella intermedia, which are key periodontal pathogens [11,12]. But, the long-term use of chlorhexidine mouthwash could present some side effects: taste alteration, numbness and pain in mouth and tongue, xerostomia, and subjective discoloration [13,14]. Thus, besides traditional solutions, researchers are focusing on alternative therapies that do not include topical antibiotics. Among the various substances studied, ozone seems to be effective [15]. Ozone is a powerful oxidant used in dentistry as a gas, ozonated water, or oil mixtures. It has antimicrobial, immunostimulant, and biosynthetic effects, effectively destroying microorganisms by oxidizing their membranes and proteins, which are essential in dentistry [16,17]. In fact, in dentistry, ozone is used to disinfect and treat various oral conditions, from periodontal pocket irrigation to reducing tooth sensitivity [18]. Studies have shown promising results of ozone use against caries and periodontitis, but further research is needed to standardize treatment protocols [19,20]. The solubilized form of ozone is necessary for its stability, and olive oil could serve as a carrier due to its reaction with monounsaturated fatty acids, leading to the production of ozonide, aldehydes, peroxides, and hydroxy peroxides, which also enhance ozone’s antimicrobial activity, demonstrating its antibacterial properties in adjunctive periodontitis treatment [21,22]. As well as ozone, another proposed support therapy for periodontitis is probiotic supplement therapy. Probiotics are nonpathogenic live microorganisms that improve the host’s health and can re-establish a healthy microbiome [23]. Some promising results have been reported for inhibiting periodontal pathogens, with direct clinical benefits in improving gingival index and bleeding on probing scores [24,25]. Researchers have also demonstrated how prebiotics and probiotics could decrease pathogenic oral bacteria and decrease the initiation and progression of periodontal disease in human clinical studies [26]. However, the use of probiotics and prebiotics in treatment remains experimental and is not yet included in official guidelines [2]. Few clinical studies [8,22,27] have investigated whether a protocol using selective antiseptics, such as ozonated olive oil, or probiotics [28,29] can effectively promote eubiotic microbiota in the maintenance therapy of periodontitis, and the eventual relationship between oral biofilm composition and the species represented within all the oral microbiota during domiciliary procedures remains under debate. This study aims to investigate the clinical benefits of combining ozonated olive oil products (mouthwash and toothpaste) with probiotics on oral health status in patients with stage I and II periodontitis following the active phase of therapy.

## 2. Materials and Methods

### 2.1. Protocol

The study was conducted in accordance with the Declaration of Helsinki of 1965, revised in Tokyo in 2004. It was registered on clinicaltrial.gov (Name of the registry: Probiotics And Ozonated Olive Oil To Maintain Oral Eubiosis In Stage I And II Periodontitis Patients, Trial registration number: NCT06955546, Date of registration: 27 May 2025) and received local ethical committee approval (Prot. 1983/CEL of 16 December 2024) before patient enrollment.

A randomized clinical trial was conducted from July to November 2025 in the Department of Dentistry and Stomatology of the University Hospital “Policlinico di Bari”, 70124, Bari (BA), South Italy. The patients were subsequently randomized into three parallel groups (A, B, or C) at the end of the professional periodontal therapy. The protocol was a triple-blind, randomized and controlled trial (RCT) with three parallel arms, lasting 40 days per enrolled patient, and encompassing both diagnostic and operational phases.

1. Identification of the reference population: the study included only patients who required professional therapy for periodontal disease, classified as stage 1 or 2 according to the 2017 classification of periodontal diseases [2].
2. Randomization: a software (Research Randomizer, by Geoffrey C. Urbaniak and Scott Plous, Version 4.0, available at https://www.randomizer.org, accessed on 8 October 2025) generated a randomization list (A, B, C) provided by the study coordinator that was used to assign patients to the study groups at the end of the active phase.
3. Allocation: Individuals involved in the clinical work, as active therapy providers or parameter evaluators, were unaware of the type of treatment administered to the patient. Identical packaging was prepared for mouthwash, toothpaste, and probiotic tablets, identifiable only by barcodes corresponding to the assignment group (A, B, C), which was known only to the study coordinator. Patient assignment was determined by opening the envelope containing the barcode.
4. Blinding: A single-blinded investigator/evaluator collected all data for the study. The evaluator could confer with the active therapy provider, provided that the latter remained unaware of the type of product delivered. To ensure blinding, the key to the anonymized products was kept by the study coordinator until the end of the study. Patients were also unaware of their group assignment. The statistician and the sponsor liaison (RE) were not informed of the specific patient group assignments until the study and statistical analysis were completed.

The TREND checklist was completed and can be found in Appendix A.

### 2.2. Sampling

The selection criteria were as follows.

The researcher included those who met the following inclusion criteria:

- Male or female, aged between 18 and 70 years, of any race;
- Diagnosis of stage I or II periodontitis according to the American Academy of Periodontology and the European Federation of Periodontology 2017 classification of periodontal diseases;
- Good general health;
- Ability to understand and comprehend the study’s instructions and sign the informed consent form.

The main exclusion criteria were:

- Pregnancy and breastfeeding;
- Periodontal or antibiotic therapy in the last two months;
- Systemic diseases that could influence the severity of periodontal disease or therapeutic success (e.g., Down syndrome, HIV, and Diabetes Mellitus);
- Smoking > 10 cigarettes per day;
- Need for antibiotic prophylaxis for dental procedures;
- Chronic use of anti-inflammatory drugs, calcium channel blockers, antidepressants, and antiepileptics.

### 2.3. Arms Description

Each patient was randomly assigned to one of three arms of this study during the T1 visit/active phase with a specialized online Software (Research Randomizer, by Geoffrey C. Urbaniak and Scott Plous, Version 4.0, available at: https://www.randomizer.org, accessed on 8 October 2025). Only the study coordinator (CO) was aware of the relationship between each individual and their belonging group.

**Group A: placebo group**

In this group, the patients received a placebo toothpaste, mouthwash, and probiotics. Their appearance and taste were kept quite similar to mimic the real products containing effective substances to dissuade the patient from dropping out. The excipients were retained; the active ingredients were excluded.

**Group B: effective toothpaste and mouthwash, placebo probiotics**

The patients of this group received effective toothpaste (Curasept Prevent toothpaste^®^, Saronno, 21047, Italy) and mouthwash (Curasept Prevent Mouthwash^®^, Saronno, 21047, Italy).

**Group C: effective toothpaste, mouthwash, and probiotics**

The patients of this group received effective toothpaste (Curasept Prevent toothpaste^®^, Saronno, 21047, Italy), mouthwash (Curasept Prevent Mouthwash^®^, Saronno, 21047, Italy), and probiotics (Curasept Prevent probiotics^®^, Saronno, 21047, Italy).

**Figure 1:**
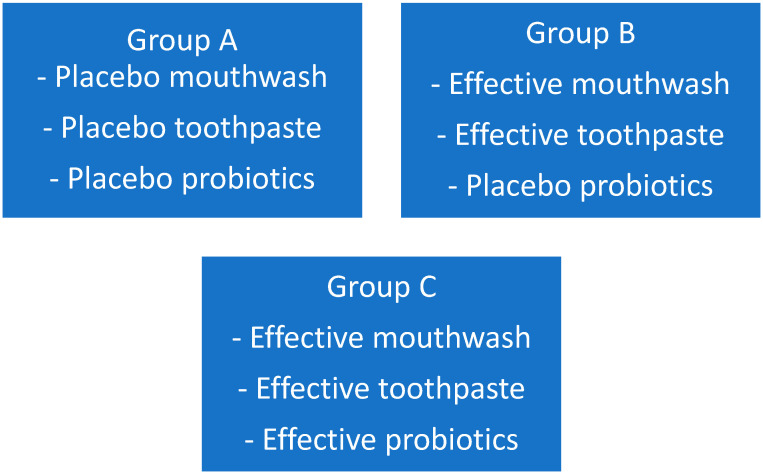
Synoptic figure of the three arms.

The three groups are described in the following graph (Figure 1).

### 2.4. Operational Sequence

Each patient underwent three visits, with an additional visit dedicated to the active phase when needed. Before enrollment, informed consent was obtained, and the study’s objectives, the risks and benefits of the therapies, and the research purpose were provided (including clinical procedures, follow-up visits, and potential risks).

The actions needed for all timepoints are described as follows:

- T0 Screening Visit: Inclusion and exclusion criteria were identified. During this visit, the demographic variables were recorded in the first periodontal chart. If the patient can be enrolled, they will receive standardized domiciliary oral hygiene instructions. Toothbrushes and interdental brushes were Curasept Soft 012^®^ and Curasept Proxi^®^ (Curasept, Saronno, 21047, Italy), respectively. Clinicians took initial photographs.
- T1 Active Visit: After 10 days from the T0 Screening Visit, two expert clinicians evaluated the adherence to the hygiene instructions given at T0, recording further study variables and completing the periodontal chart. Non-surgical periodontal therapy was performed within 24 h by a single-blind operator. Supragingival and subgingival scaling and root planning were performed using manual instruments (Gracey’s curettes) and/or ultrasonic inserts (Cavitron^®^, Dentsply Sirona, Charlotte, NC, USA). Finally, each patient was randomly assigned to one of the three study groups, and the study coordinator gave them toothpaste, mouthwash, and probiotics. The toothpaste was used twice daily, in the morning and evening, followed by rinsing with undiluted mouthwash for 1 min. After rinsing, the patient did not rinse further with water and refrained from eating, drinking, and smoking for at least one hour. Domiciliary maneuvers and probiotic assumption were continued, without modification, for 30 days.
- T2 Follow-Up Visit: This visit was conducted at 30 days after the T1 Active Visit. During the visit, adherence was checked, and any adverse events or side effects of the provided therapy were recorded. The study variables were measured again, and the periodontal chart was completed. Final photographs were taken. At the end of the 30th day, the clinical protocol was considered completed.
- Drop-Outs: Each patient could drop out at any time. All cases of study withdrawal were recorded in the patient’s medical record. If a patient withdrew from the study before the follow-up visit, the data were considered invalid, and the patient was excluded.

### 2.5. Outcome Evaluation

The single-blind evaluator records the following clinical parameters during each visit:

- PPD (Probing Pocket Depth): The depth of the pocket at four sites per tooth, measured from the free gingival margin to the base of the pocket using a UNC15 periodontal probe. Periodontal pockets were classified as moderate (PPD = 4–6 mm) or deep (PPD ≥ 7 mm) and analyzed as subgroups.
- The Full-Mouth Plaque Score (FMPS) and Full-Mouth Bleeding Score (FMBS) were percentage indices based on dichotomous recordings (absence/presence of plaque or bleeding, respectively) at four sites per tooth, evaluating the patient’s oral hygiene and inflammation status.
- Adherence: The quality of adherence to prescribed therapies was verified at each visit. Adherence was assessed as “valid” or “invalid.”

A synoptic table of the clinical procedures is reported as follows (Table 1).

### 2.6. Statistical Analysis

For the continuous baseline characteristic (age) and patient outcomes (measured at T1, T2, and as the difference between T2 and T1 [delta T2-T1]), normality was assessed using the Shapiro–Wilk test. As these variables did not follow a normal distribution, they were summarized as medians and interquartile ranges (IQRs). Comparisons of these parameters between patient cohorts (groups A, B, and C) were performed using the Kruskal–Wallis test. Changes within groups, pre- and post-treatment, were analyzed using a repeated-measures analysis of variance (ANOVA).

Categorical parameters, including sex and periodontal stage, were reported as absolute and relative frequencies and compared using the Chi-square test. A rank-based general linear model (Rank-GLM) was applied to evaluate the effect of treatment group on delta outcomes (T2-T1), as these differences did not meet the assumption of normality. Delta outcomes were transformed into ranks before analysis, and the model included age, sex, and periodontal stage as covariates. Pairwise comparisons of the delta estimate between groups were adjusted using Tukey’s method. A *p*-value < 0.05 was considered statistically significant.

The sample size determination was performed through a prior power analysis for a one-way ANOVA with three groups. A medium effect size (f = 0.45) was chosen, which represents a moderate effect and is considered clinically relevant for evaluating differences in the Full-Mouth Plaque Score (FMPS) between treatment groups. Although no specific studies are available comparing the treatments in this study, the selected effect size was based on previous studies analyzing the efficacy of non-surgical periodontal therapy and the low intra-study variability typically reported for this clinical parameter, which allows for the detection of differences even with a moderate effect [8,22,30].

With a significance level of 5% and a statistical power of 80%, the sample size calculation yielded 18 participants per group (a total of 54 subjects). However, to account for an estimated 10% drop-out rate, enrollment was planned to include 20 patients per group, resulting in a final total of 60 participants. The power calculation was performed using the G*Power software (Version 3.1.9.4).

The null hypothesis was that there was no statistical difference among the three groups.

All analyses were conducted using SAS/STAT^®^ version 9.4 (SAS Institute, Cary, NC, USA).

## 3. Results

Of 89 screened patients, 63 patients were enrolled who respected the inclusion criteria and were equally divided into the three groups. The median age was 53 years [IQR 44–59], with no significant differences observed among the three groups. Females accounted for 54% of the sample, with no significant differences between groups.

Regarding the periodontal stage, 74.6% of the patients were classified as Stage II, distributed as follows: 66.7% in group A, 71.4% in group C, and 85.7% in group B. However, these differences were not statistically significant. There are some discrepancies at the baseline, which depend on the individual’s response at the active visit and the individual adherence to the domiciliary oral hygiene instructions.

The FMPS percentages at both T1 and T2 were significantly different among the three groups (*p* = 0.003 and *p* < 0.0001, respectively). A 34% reduction was observed from T1 to T2, with a statistically significant difference between the groups (*p* = 0.0002). Specifically, group A showed a reduction of 24% [IQR −42; −12], group B a decrease of 33% [IQR −42; −27], and group C a reduction of 62% [IQR −70; −40].

Similarly, the FMBS percentages were significantly different among the groups at both the initial (T1) visit (*p* = 0.0058) and at the follow-up visit (T2) (*p* < 0.0001). The most significant reduction was observed in group C (−49% [IQR −61; −42]) compared with group B (−20% [IQR −26; −15]) and group A (−15% [IQR −24; −11]) (*p* < 0.0001).

The mean PPD did not differ significantly among the groups at T1 (*p* = 0.206) but became significantly different at T2 (*p* = 0.0013). Consequently, the reduction in PPD from T1 to T2 also showed highly significant differences among the groups (*p* < 0.0001). Patients in group C exhibited the greatest decrease (−1.10 mm [IQR −1.30; −0.60]) compared to smaller reductions in group A (−0.10 mm [IQR −0.20; −0.10]) and group B (−0.40 mm [IQR −0.50; −0.20]). In all three outcomes, the reductions observed at T2 were statistically significant, as indicated by the interquartile range (IQR), with both quartiles consistently negative.

Overall, group C showed the best improvement of clinical indexes between T1 and T2, followed by groups B and A.

A synoptic table is reported as follows (Table 2).

**Figure 2:**
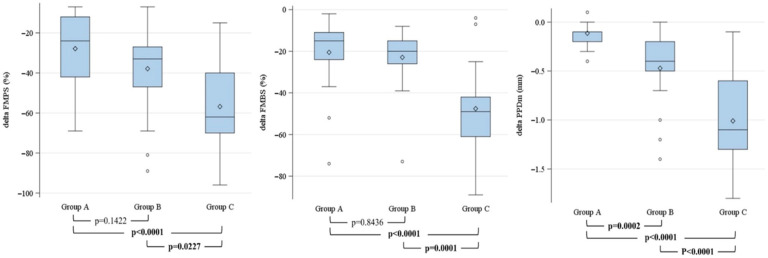
Differences in FMPS, FMBS, and mean PPD reductions among the three treatment groups estimated using rank-based general linear models.

Figure 2 illustrates the results of the rank-based general linear models applied to evaluate differences in the reductions in FMPS, FMBS, and mean PPD across the three treatment groups, adjusted for age, sex, and periodontal stage (Figure 2).

In group C, the reductions in FMPS and FMBS percentages were significantly greater than in groups A and B. In contrast, no significant difference was observed between groups A and B. Conversely, the reduction in mean PPD was significantly greater in group B compared to group A, and in group C compared to both groups A and B.

In all patients, regardless of treatment group, adherence to treatment was satisfactory.

## 4. Discussion

This triple-blind, randomized and controlled trial aims to assess the clinical benefits of ozonated-olive-oil-based products (toothpaste and mouthwash) and a daily probiotic supplement in improving oral health status in patients with stage I and II periodontitis after non-surgical periodontal therapy.

The null hypothesis was rejected: there are significant differences among the three groups, which are discussed below.

Group B (effective toothpaste and mouthwash) showed an improvement in the clinical parameters when compared to group A (placebo group): the FMPS percentage was 23% (lower if compared with 50% for group A) and the FMBS percentage was 16% (lower if compared with 43% for group A), while the PPD showed no improvement. These results agree with previous authors, who demonstrate the selective efficacy of ozonated olive oil as an adjunctive treatment in non-surgical therapies for periodontitis [31,32,33]. The significant improvements of group B over the placebo validate ozonated olive oil as an adjunctive tool in periodontal maintenance. Its mechanism relies on the rapid generation of ozone derivatives (ozonide and peroxides) on periodontal tissues, resulting in a powerful non-specific antimicrobial effect [34]. In fact, in vitro data from Pietrocola et al. confirm that ozonated olive oil is an antibacterial and antifungal agent against a wide spectrum of oral pathogens implicated in periodontitis, highlighting its ability to disrupt established microbial biofilms via cell wall oxidation [35]. Moreover, ozonated water can kill Gram-positive and Gram-negative oral bacteria, as well as oral candida albicans, by inhibiting the growth of pathogenic microorganisms in dental plaque [36].

On the other hand, it has a strong anti-inflammatory effect, which may explain the significant reduction in FMBS: ozonated-olive-oil-based mouthwash led to a significant decrease in salivary matrix metalloproteinase-8 (MMP-8) levels [37]. MMP-8 is an established biomarker for inflammatory tissue destruction, and its reduction suggests that ozonated olive oil actively interferes with the host’s destructive inflammatory cascade, adding these properties to the antimicrobial one [27].

Our results are consistent with other randomized clinical trials, which have established that adjunctive ozonated olive oil can be clinically effective, comparable to chlorhexidine, which remains the gold standard [8,22,30]. Furthermore, some authors demonstrated that ozonated olive oil was significantly superior to plain olive oil, isolating the therapeutic effect to the ozone compound [38,39]. Based on this evidence, ozonated olive oil could provide a viable, safe, and effective alternative to chlorhexidine for long-term supportive therapy, given chlorhexidine’s contraindication for long-term use and its well-known side effects (discoloration, taste alteration) that could compromise patient compliance [13,30].

Group C (effective toothpaste, mouthwash, and probiotics) showed better outcomes than the other groups across all measured clinical parameters, including Full-Mouth Plaque Score (FMPS), Full-Mouth Bleeding Score (FMBS), and Probing Pocket Depth (PPD).

In fact, group C was associated with a significant reduction (*p* = 0.0002 and <0.0001, respectively) in FMPS and FMBS percentage. The FMPS percentage was 4% (lower than 23% for group B and 50% for group A) and the FMBS percentage was 2% (lower than 16 for group B and 43% for group A). Thus, the treatment given for this group was more effective in removing plaque and controlling gingival bleeding and inflammation. This finding is consistent with many authors, who reported a reduction in bleeding on probing (BOP) indexes after a probiotic administration in adjunctive non-surgical therapy [40,41,42]. This could be explained because of the significant reduction in the *P. gingivalis* count mediated by probiotics, resulting in a reduction in pro-inflammatory cytokines and nitric oxide (NO) stress among the subgingival microbiota 10.1002/ibd.20448. The addiction of probiotics to ozonated olive oil could have a pharmacological multiplier effect by preventing bacterial recolonization of the pocket following an initial knockout [43,44].

In fact, the periodontal probing depth (PPD) of group C showed a statistically significant reduction at the T2 follow-up (*p* = 0.0013), followed by group B and group A, which showed only a slight reduction. PPD reduction was 1.10 mm in group C, which is a significantly better outcome than 0.40 mm in group B and 0.10 mm in group A. This difference strongly suggests that the synergy between chemical oxidation and biological modulation could play a pivotal role in achieving long-term therapeutic advantage over traditional monotherapies, thereby improving the clinical course of chronic periodontitis. The mean PPD observed in the T2 visit of 1.8 mm [Range: 1.6–2.0] is considered to be within the physiological range, as described by Lang in their paper [45].

The most significant clinical outcome is the PPD reduction observed in group C, which could be attributed only to the synergistic action of the probiotic supplement when combined with ozonated olive oil. This finding addresses a critical gap in traditional supportive therapy: the early microbial recolonization that occurs after active treatment. While ozonated olive oil could lead to an initial microbial ‘knockdown’, the continuous use of probiotics (likely *Lactobacillus* and *Bifidobacterium*) introduces beneficial bacteria that compete with residual periodontal pathogens. Several studies have been conducted on this topic, and their results are consistent: many authors reported that the use of *Lactobacillus reuteri* as an adjunct to non-surgical periodontal therapy led to a considerable reduction in probing depth and bleeding on probing compared with the placebo [46,47]. Furthermore, a systematic review confirms that probiotics can improve clinical outcomes in patients with periodontitis, suggesting a beneficial effect on periodontal health [48]. This competitive inhibition and site-blocking mechanism stabilizes the microbiological environment of the periodontal pockets [49]. The proposed combined protocol could shift to an oral eubiosis, which may prevent the re-establishment of a pathogenic biofilm, resulting in superior and more stable reductions in PPD and FMBS [50]. Finally, adjunctive probiotics enhance non-surgical periodontal therapy outcomes, interfering with pathogen growth on one hand and modulating local immune responses on the other hand [24]. The dual benefits of initial chemical disruption mediated by ozonated olive oil followed by biological stabilization with probiotics are essential for maximizing clinical stability during the maintenance phase [51]. However, the most recent guidelines of the Italian society of periodontology note that, while promising, the current scientific evidence is insufficient to endorse the routine use of probiotics in periodontal disease treatment [2]. In addition, the EFP does not recommend the assumption of probiotics as an adjunctive therapy of stage I–III periodontitis in their guidelines [3].

### Limitations and Future Directions

Despite the strengths of this controlled and blinded study design, several limitations are present:

- This study provides a small sample of 63 patients, which does not allow for drawing definitive conclusions. Further studies with a larger sample are mandatory to confirm our findings.
- Short-term follow-up: The 30-day observation period is sufficient to observe acute changes in inflammatory indices, but it is inadequate to assess the crucial long-term stability of PPD gains or the actual efficacy of the combined protocol in preventing disease recurrence. Further research must have extended follow-up periods (e.g., 3, 6, and 12 months) to confirm the sustained therapeutic benefits.
- Another critical limitation is the absence of molecular microbiological data (e.g., quantitative PCR or next-generation sequencing) on the subgingival biofilm to confirm how ozonated olive oil influences the establishment of beneficial probiotic strains or to determine which synergistic action most effectively suppresses specific pathogenic species.

Future studies should establish a clear correlation between clinical parameters and shifts in microbial community structure.

## 5. Conclusions

The findings of this clinical trial support the use of a combined regimen of ozonated-olive-oil-based products and probiotic supplements as an adjunctive home care strategy for patients affected by stage I and II periodontitis. The antimicrobial and anti-inflammatory effects of ozonated olive oil, along with the probiotic modulation, could provide synergistic cooperation, improving all clinical outcomes in the maintenance therapy. To fully validate this promising therapeutic strategy, future trials with larger samples must focus on long-term follow-up and should include detailed molecular and microbiological analyses.

## Figures and Tables

**Table 1:** Summary of clinical procedures.

Procedures	Screening (T0)	Active Phase (T1)	Follow-Up (T2)
	Day-7	Day 0	Day 30
Inclusion and Exclusion Criteria	**✓**		
“Informed Consent Delivery and Explanation”	**✓**		
Photographs	**✓**		**✓**
Signature of Informed Consent	**✓**		
Standardized Oral Hygiene Instructions	**✓**	**✓**	
Delivery of Toothbrush and Interdental Brush	**✓**		
FMPS (Full-Mouth Plaque Score)	**✓**	**✓**	**✓**
FMBS (Full-Mouth Bleeding Score)	**✓**	**✓**	**✓**
PPD (Periodontal Probing Depth)	**✓**		**✓**
Active Phase of Periodontal Therapy		**✓**	
Randomization		**✓**	
Delivery of Products Corresponding to Groups A, B, and C		**✓**	
Adherence Monitoring		**✓**	**✓**
Recording of Adverse Events			**✓**

**Table 2:** Main characteristics of patients and values for FMPS, FMBS, and mean PPD stratified by treatment group.

Parameter	All (n = 63)	Group A (n = 21)	Group B (n = 21)	Group C (n = 21)	*p*
Mean Age [Range]	53 [44–59]	54 [42–58]	52 [45–59]	54 [44–63]	0.9210
Sex					
F (%)	34 (54.0%)	11 (52.4%)	11 (52.4%)	12 (57.1%)	0.9381
M (%)	29 (40.0%)	10 (47.6%)	10 (47.6%)	9 (42.9%)	
Periodontitis Stage					
I (%)	16 (25.4%)	7 (33.3%)	3 (14.3%)	6 (28.6%)	0.3365
II (%)	47 (74.6%)	14 (66.7%)	18 (85.7%)	15 (71.4%)	
Mean FMPS					
T1% [Range]	69 [58–80]	78 [70–88]	61 [55–72]	68 [57–75]	**0.0030**
T2% [Range]	22 [5–42]	50 [35–69]	23 [20–32]	4 [2–8]	**<0.0001**
Delta (T2-T1)	−34 [−62;−24]	−24 [−42;−12]	−33 [−47;−27]	−62 [−70;−40]	**0.0002**
Mean FMBS					
T1% [Range]	51 [31–63]	61 [40–70]	38 [27–44]	56 [42–63]	**0.0058**
T2% [Range]	10 [2–22]	43 [17–48]	16 [6–19]	2 [0–5]	**<0.0001**
Delta (T2-T1) [Range]	−24 [−47;−14]	−15 [−24;−11]	−20 [−26;−15]	−49 [−61;−42]	**<0.0001**
Mean PPD					
T1 mm [Range]	2.1 [1.8–2.8]	1.9 [1.8–2.2]	2.2 [2.0–2.5]	2.5 [1.7–2.9]	0.2060
T2 mm [Range]	1.8 [1.6–2.0]	1.8 [1.7–2.0]	1.8 [1.6–2.0]	1.3 [1.2–1.8]	**0.0013**
Delta (T2-T1) [Range]	−0.4 [−0.9;−0.1]	−0.1 [−0.2;−0.1]	−0.4 [−0.5;−0.2]	−1.1 [−1.3;−0.6]	**<0.0001**

**Figure A1:**
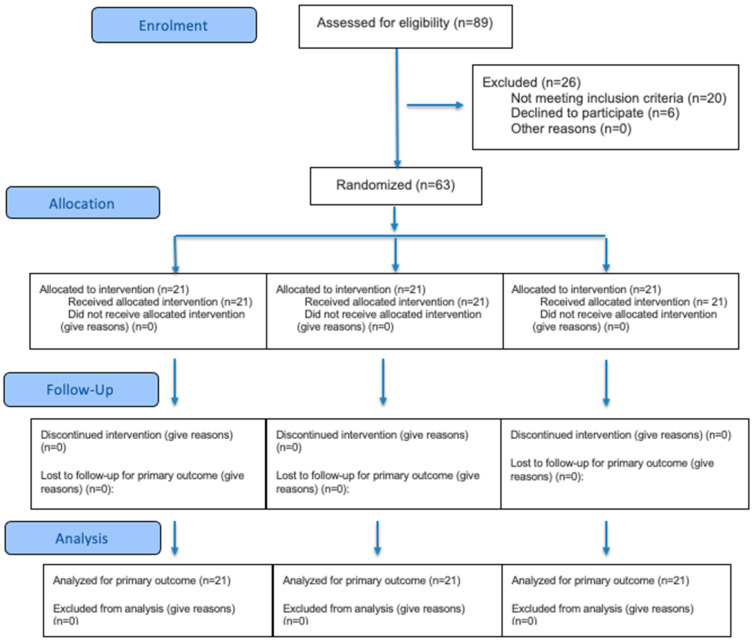
CONSORT 2025 Diagram.

## Data Availability

The original contributions presented in this study are included in the article. Further inquiries can be directed to the corresponding author.

## Appendix A

**Table A1:** CONSORT 2025 Checklist.

Section/Topic	No	CONSORT 2025 Checklist Item Description	Reported on Page No.
**Title and abstract**			
Title and structured abstract	1a	Identification as a randomized trial	1
	1b	Structured summary of the trial design, methods, results, and conclusions	1
**Open science**			
Trial registration	2	Name of trial registry, identifying number (with URL) and date of registration	3
Protocol and statistical analysis plan	3	Where the trial protocol and statistical analysis plan can be accessed	3–7
Data sharing	4	Where and how the individual de-identified participant data (including data dictionary), statistical code and any other materials can be accessed	No
Funding and conflicts of interest	5a	Sources of funding and other support (e.g., supply of drugs), and role of funders in the design, conduct, analysis and reporting of the trial	No
	5b	Financial and other conflicts of interest of the manuscript authors	No
**Introduction**			
Background and rationale	6	Scientific background and rationale	2–3
Objectives	7	Specific objectives related to benefits and harms	3
**Methods**			
Patient and public involvement	8	Details of patient or public involvement in the design, conduct and reporting of the trial	3–4
Trial design	9	Description of trial design including type of trial (e.g., parallel group, crossover), allocation ratio, and framework (e.g., superiority, equivalence, non-inferiority, exploratory)	3
Changes to trial protocol	10	Important changes to the trial after it commenced including any outcomes or analyses that were not prespecified, with reason	No
Trial setting	11	Settings (e.g., community, hospital) and locations (e.g., countries, sites) where the trial was conducted	3
Eligibility criteria	12a	Eligibility criteria for participants	4
	12b	If applicable, eligibility criteria for sites and for individuals delivering the interventions (e.g., surgeons, physiotherapists)	No
Intervention and comparator	13	Intervention and comparator with sufficient details to allow replication. If relevant, where additional materials describing the intervention and comparator (e.g., intervention manual) can be accessed	4–5
Outcomes	14	Pre-specified primary and secondary outcomes, including the specific measurement variable (e.g., systolic blood pressure), analysis metric (e.g., change from baseline, final value, time to event), method of aggregation (e.g., median, proportion), and time point for each outcome	6
Harms	15	How harms were defined and assessed (e.g., systematically, non-systematically)	6–7
Sample size	16a	How sample size was determined, including all assumptions supporting the sample size calculation	6–7
	16b	Explanation of any interim analyses and stopping guidelines	No
Randomisation			
Sequence generation	17a	Who generated the random allocation sequence and the method used	4
	17b	Type of randomisation and details of any restriction (e.g., stratification, blocking and block size)	No
Allocation concealment mechanism	18	Mechanism used to implement the random allocation sequence (e.g., central computer/telephone; sequentially numbered, opaque, sealed containers), describing any steps to conceal the sequence until interventions were assigned	4
Implementation	19	Whether the personnel who enrolled and those who assigned participants to the interventions had access to the random allocation sequence	No
Blinding	20a	Who was blinded after assignment to interventions (e.g., participants, care providers, outcome assessors, data analysts)	3
	20b	If blinded, how blinding was achieved and description of the similarity of interventions	No
Statistical methods	21a	Statistical methods used to compare groups for primary and secondary outcomes, including harms	6–7
	21b	Definition of who is included in each analysis (e.g., all randomized participants) and in which group	4
	21c	How missing data were handled in the analysis	No
	21d	Methods for any additional analyses (e.g., subgroup and sensitivity analyses), distinguishing prespecified from post hoc	No
**Results**			
Participant flow, including flow diagram	22a	For each group, the numbers of participants who were randomly assigned, received intended intervention, and were analyzed for the primary outcome	7–8
	22b	For each group, losses and exclusions after randomization, together with reasons	No
Recruitment	23a	Dates defining the periods of recruitment and follow-up for outcomes of benefits and harms	5
	23b	If relevant, why the trial ended or was stopped	No
Intervention and comparator delivery	24a	Intervention and comparator as they were actually administered (e.g., where appropriate, who delivered the intervention/comparator, how participants adhered, whether they were delivered as intended [fidelity])	3–4
	24b	Concomitant care received during the trial for each group	No
Baseline data	25	A table showing baseline demographic and clinical characteristics for each group	8
Numbers analyzed, outcomes and estimation	26	For each primary and secondary outcome, by group: the number of participants included in the analysis the number of participants with available data at the outcome time point result for each group, and the estimated effect size and its precision (such as 95% confidence interval) for binary outcomes, presentation of both absolute and relative effect size	7–8
Harms	27	All harms or unintended events in each group	No
Ancillary analyses	28	Any other analyses performed, including subgroup and sensitivity analyses, distinguishing pre-specified from post hoc	No
**Discussion**			
Interpretation	29	Interpretation consistent with results, balancing benefits and harms, and considering other relevant evidence	9–11
Limitations	30	Trial limitations, addressing sources of potential bias, imprecision, generalizability, and, if relevant, multiplicity of analyses	10–11
